# In Situ Electrospun Porous MIL-88A/PAN Nanofibrous Membranes for Efficient Removal of Organic Dyes

**DOI:** 10.3390/molecules28020760

**Published:** 2023-01-12

**Authors:** Hao Wu, Le Xu, Jiao Jia, Fengchun Dong, Yongtang Jia, Xi Liu

**Affiliations:** Guangdong-Hong Kong Joint Laboratory for New Textile Materials, Guangdong Functional Fiber and Textile Engineering Technology Research Center, School of Textile Materials and Engineering, Wuyi University, Jiangmen 529020, China

**Keywords:** electrospun, nanofibrous membranes, MIL-88A, dye adsorption and removal

## Abstract

In recent years, metal–organic framework (MOF)-based nanofibrous membranes (NFMs) have received extensive attention in the application of water treatment. Hence, it is of great significance to realize a simple and efficient preparation strategy of MOF-based porous NFMs. Herein, we developed a direct in situ formation of MOF/polymer NFMs using an electrospinning method. The porous MOF/polymer NFMs were constructed by interconnecting mesopores in electrospun composite nanofibers using poly(vinylpolypyrrolidone) (PVP) as the sacrificial pore-forming agent. MOF (MIL-88A) particles were formed inside the polyacrylonitrile (PAN)/PVP nanofibers in situ during electrospinning, and the porous MIL-88A/PAN (pMIL-88A/PAN) NFM was obtained after removing PVP by ethanol and water washing. The MOF particles were uniformly distributed throughout the pMIL-88A/PAN NFM, showing a good porous micro-nano morphological structure of the NFM with a surface area of 143.21 m^2^ g^−1^, which is conducive to its efficient application in dye adsorption and removal. Specifically, the dye removal efficiencies of the pMIL-88A/PAN NFM for amaranth red, rhodamine B, and acid blue were as high as 99.2, 94.4, and 99.8%, respectively. In addition, the NFM still showed over 80% dye removal efficiencies after five adsorption cycles. The pMIL-88A/PAN NFM also presented high adsorption capacities, fast adsorption kinetics, and high cycling stabilities during the processes of dye adsorption and removal. Overall, this work demonstrates that the in situ electrospun porous MOF/polymer NFMs present promising application potential in water treatment for organic dyestuff removal.

## 1. Introduction

Dyes, pesticides, food additives, pharmaceuticals, personal care products, and other emerging organic contaminants in water resources present numerous adverse effects on human health and ecosystem [1]. Owing to the development of industries, such as the textile dyeing and finishing industry, the amount of organic dyes polluting industrial wastewater has increased [1,2,3,4]. Therefore, researchers have focused on developing adequate wastewater treatment methods [5,6]. To date, numerous physical and chemical methods, such as advanced oxidation [7], adsorption [8,9,10,11,12,13], coagulation-flocculation [14], and photocatalysis [15], have been used for organic dye remediation. Of these, adsorption has been one of the most cost-effective methods owing to its simplicity and low energy consumption [16,17,18,19]. Therefore, it is critical to develop novel materials with high adsorption capacities for the efficient removal of organic dyes from wastewater.

Metal–organic frameworks (MOFs), owing to their diverse structure, high surface area, and tunable pore size, have been widely used in gas storage and separation applications and organic molecules adsorbents [20,21,22,23,24,25,26]. In particular, MOFs with accurate chemical design have been successfully used to adsorb, separate, and remove organic ionic dyes [24,27]. For example, Li et al. prepared a MOF-based nanofiber filter using an electrostatic spinning method and used it to adsorb and selectively separate cationic dyes from aqueous solutions [28]. Jhung et al. prepared urea- or melamine-modified MIL-100(Cr), which is a highly efficient adsorbent for nitroimidazole dyes [29]. However, the applications of powdered MOF particles in organic dye treatment of continuous wastewater are relatively limited [30]. Therefore, it is crucial to develop novel methods for the preparation of polymer–MOF composite membranes for efficient and stable treatment of organic dyes in continuous fluid wastewater.

Electrospinning is a fiber fabrication method used to produce long and continuous polymer fibers with diameters in the nano- or micrometer ranges [31,32,33,34,35,36,37,38]. Furthermore, the fibers form stacks during electrospinning, yielding nonwoven membranes [33,38]. Owing to their high porosity and easy preparation, electrospun polymer fibers have recently been integrated with MOFs to shape MOFs into fibers [29,30,31,32]. For example, Zhao et al. proposed a simple method for the uniform growth of porous MIL-100(Fe) on electrospun polyacrylonitrile (PAN) fiber membranes via electrospinning and hydrothermal reactions [38]. Similarly, Lu et al. deposited UiO-66-NH_2_ on the surface of electrospun PVDF–MOF linker nanofibrous membranes (NFMs) and used the composite NFMs for removal of toxic chemicals [39]. Xie et al. prepared a graphene oxide (GO)/MIL-88A(Fe) membrane by embedding MIL-88A into a GO matrix. The membrane exhibited remarkable recovery and self-cleaning performance and excellent degradation performance for organic pollutants under ultraviolet (UV) irradiation [40]. Leus et. al. embedded Pt@MIL-101 into a poly-ε-caprolactone matrix via electrospinning to create a “catalytic carpet” [41]. The current methods used to prepare polymer–MOF composite NFMs can be classified as follows: (1) hydrothermal synthesis followed by loading MOFs onto the surface of the NFMs after spinning and (2) direct blending of MOFs with polymer solutions for spinning [28]. These processes are complex; moreover, pure MOF particles are not dispersed uniformly throughout the polymer matrix during spinning [28,42]. Therefore, it is critical to develop new methods to fabricate polymer–MOF composite NFMs.

In this study, we developed a direct in situ formation of MOF/polymer NFM using an electrospinning method. Moreover, the porous MOF/polymer NFMs were constructed by interconnecting mesopores in electrospun composite nanofibers using poly(vinylpolypyrrolidone) (PVP) as the sacrificial pore-forming agent. We designed and prepared MIL-88A powder and developed a simple one-step electrospinning method for in situ binding MIL-88A onto PAN nanofibers. Specifically, MIL-88A MOF particles were formed inside the PAN/PVP fibers in situ during electrospinning, and porous MIL-88A/PAN (pMIL-88A/PAN) NFM was obtained after removing PVP by ethanol and water washing. The MOF particles were uniformly distributed throughout the pMIL-88A/PAN NFM, showing a good porous micro-nano morphological structure of the NFM, which is conducive to its efficient application in dye adsorption and removal. Accordingly, the pMIL-88A/PAN NFM presented high adsorption capacities, fast adsorption kinetics, and high cycling stability for the removal of amaranth red, rhodamine B, and acid blue dyes. Overall, this work provides an effective strategy for electrospinning composite MOF/polymer NFMs, which will benefit future research and applications in water treatment and other fields.

## 2. Results and Discussion

### 2.1. Preparation and Characterization of the pMIL-88A/PAN NFMs

**Figure 1:**
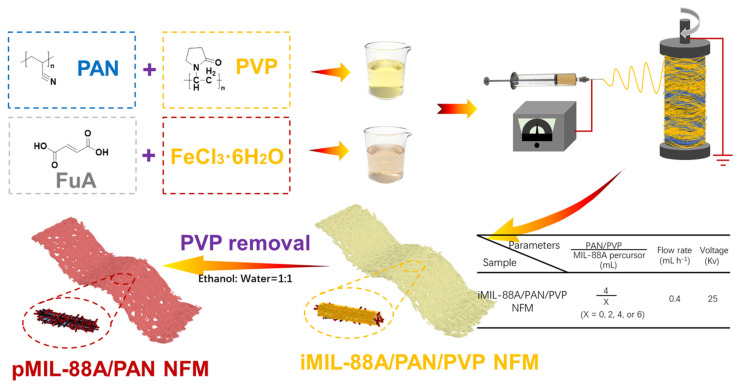
Schematic for the fabrication of pMIL-88A/PAN NFMs.

Figure 1 shows the schematic for the preparation of the porous MIL-88A/PAN (pMIL-88A/PAN) NFM. Considering the simplicity and universality of the MOF structure, an iron-based MOF (MIL-88A) formed from FuA and FeCl_3_ 6H_2_O was used in this study. In situ MOF formation, polymer solidification, and solvent evaporation occurred simultaneously during electrospinning of the spinning solution containing PAN, PVP, FuA, and FeCl_3_ 6H_2_O. Owing to the difference in solubility, PVP was added to the PAN spinning solution as the supporting pore-forming sacrificial agent, whereas PAN served as the polymer nanofiber skeleton. Therefore, the MIL-88A combined porous nanofiber can be formed after the PVP is removed by ethanol and water washing. More importantly, the originally embedded MIL-88A particles can be regularly exposed to the surface of the porous PAN nanofibers, increasing its specific surface area, and providing abundant adsorption sites for dyes. Meanwhile, the macropores of the PAN fibers provide ample space for adsorption and decrease the mass transfer resistance for dye treatment of continuous wastewater. Overall, the proposed in situ electrospun scheme is a simple and effective strategy for the preparation of pMIL-88A/PAN NFM, and the NFM will be effectively used for the removal of organic dyes in the treatment of continuous wastewater.

**Figure 2:**
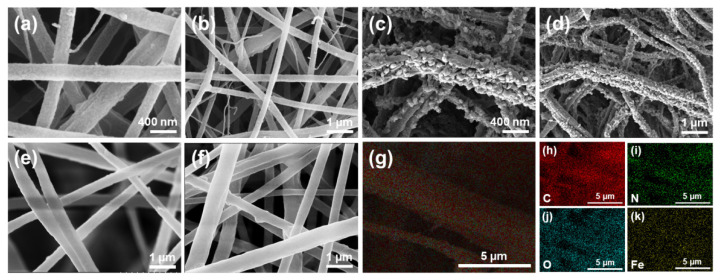
SEM images of the iMIL-88A/PAN/PVP (**a**,**b**) and pMIL-88A/PAN (**c**,**d**) NFMs with scale bars of 400 nm and 1 μm. SEM images of the iMIL-88A/PAN (**e**) and bMIL-88A/PAN (**f**) NFMs. Overall (**g**), C (**h**), N (**i**), O (**j**), and Fe (**k**) elemental mappings of a pMIL-88A/PAN NFM.

Details of the preparation of the relevant electrospun NFMs are shown in the Experimental section and Supporting Information. To confirm the formation of the NFMs, their morphology was evaluated. Figure S1a,b show the scanning electron microscopy (SEM) images of the initial PAN composite PVP (PAN/PVP) NFM, presenting uniform nanofibers with diameters of ≈300 nm as well as relatively smooth surfaces, which indicates that PAN and PVP are well mixed and fibrillar during spinning. After sufficient ethanol and water washing of PAN/PVP NFM to remove PVP, porous PAN (pPAN) NFM was obtained. Figure S1c,d shows its rough and uneven surface morphology after removing PVP, suggesting the feasibility of PVP as a supporting pore-forming sacrifice agent for the preparation of porous NFM. Then, FuA and FeCl_3_ 6H_2_O were added to PAN/PVP spinning solution to prepare in situ MIL-88A/PAN/PVP (iMIL-88A/PAN/PVP) and pMIL-88A/PAN NFMs. Since the MIL-88A particles formed in situ in the process of electrospinning are wrapped inside the fibers, iMIL-88A/PAN/PVP shows morphological characteristics similar to common nanofibers (Figure 2a,b). Therefore, the aggregation morphology of MIL-88A formed in situ can be observed in SEM images of iMIL-88A/PAN/PVP with removal of PVP, and it can be optimized by adjusting the feeding rate during electrospinning. As shown in Figure S2, less obvious MOF particles were displayed on the surface of NFM at a feeding rate as high as 0.8 mL h^−1^. With the feeding rate as low as 0.2 mL h^−1^, however, large prismatic crystal particles of MIL-88A were formed, rendering the NFM fragile, which was not conducive to further application. Through comparison, pMIL-88A/PAN NFM with more suitable morphological characteristic was achieved at a feeding rate of 0.4 mL h^−1^ than 0.6 mL h^−1^ (Figure S2b,c). Correspondingly, Figure 2c,d show the SEM images of the optimal pMIL-88A/PAN NFM with scale bars of 400 nm and 1 μm, respectively. The particles on the nanofiber membrane exhibited long hexagonal rhombic columnar crystal characteristics (Figure S3), indicating that the nanofiber membrane contains a large amount of MIL-88A particles [43,44,45,46]. Numerous homogeneous and dense particles with diameters of 50–100 nm appeared on the rough surface of fibers, confirming that MIL-88A was successfully embedded onto the surface of the PAN fibers and formed pMIL-88A/PAN nanofibers. For the purpose of comparison, in situ MIL-88A/PAN (iMIL-88A/PAN) and blended MIL-88A with PAN spinning solution (bMIL-88A/PAN) NFMs were also prepared. As shown in Figure 2e,f, the conventional and relatively smooth surface morphologies indicated that the in situ or pure MOF particles were both encapsulated inside of the PAN fibers, suggesting the significance of PVP auxiliary preparation of pMIL-88A/PAN NFM. Energy-dispersive spectroscopy (EDS) experiments were used to determine the C, N, O, and Fe distributions of the pMIL-88A/PAN NFM, and the EDS spectrum is shown in Figure S4. According to the elemental mappings of the NFM, C, N, O and Fe were well distributed throughout the surface of pMIL-88A/PAN nanofibers (Figure 2g–k), which verified the fabrication and uniform distribution of MIL-88A on the surface of PAN nanofibers. Overall, these results demonstrated that a good micro-nano morphological pMIL-88A/PAN NFM was successfully prepared by electrospinning.

**Figure 3:**
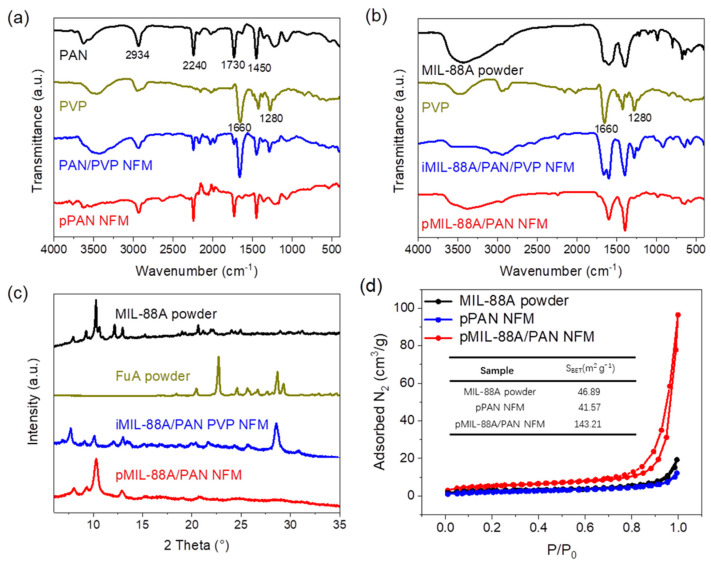
FT−IR spectra (**a**,**b**) and XRD patterns (**c**) of the relevant samples. (**d**) N_2_ adsorption−desorption isotherms of the MIL-88A powder, pPAN and pMIL-88A/PAN NFMs; BET surface areas in the inset.

The chemical structures of the as-fabricated NFMs were analyzed using FT-IR spectroscopy (Figure 3a,b). As shown in Figure 3a, the characteristic peaks at 2934, 2240, 1730, and 1450 cm^−1^ were assigned to the vibrations of the main chain and pendant group (−C≡N) of PAN, and the carbonyl and tertiary amine groups of PVP were observed with the characteristic peaks of 1660 and 1280 cm^−1^, respectively. Compared to the PAN/PVP NFM, the absence of characteristic peaks of PVP in the FT-IR spectrum of pPAN NFM indicates that PVP has been effectively and completely removed during the preparation of the porous NFMs. The similar changes of characteristic peaks of PVP in the FT-IR spectra of iMIL-88A/PAN/PVP and pMIL-88A/PAN NFMs are exhibited in Figure 3b, combined with the characteristic peaks of MIL-88A (1608, 1392, and 810–510 cm^−1^), which confirmed the formation of the chemical structure of pMIL-88A/PAN NFM. The XRD patterns of MIL-88A, FuA, iMIL-88A/PAN/PVP, and pMIL-88A/PAN NFMs are shown in Figure 3c. The peaks at 10.2° and 12.8° in the XRD patterns were ascribed to the (101) and (002) crystal planes of MIL-88A [42], which were present in the XRD patterns of iMIL-88A/PAN/PVP and pMIL-88A/PAN NFMs, indicating that MIL-88A particles were incorporated into the NFMs. The peak at 28.5° of iMIL-88A/PAN/PVP NFM may be attributed to the redundant FuA in the NFM, whereas this peak in pMIL-88A/PAN NFM disappeared due to ethanol and water rinsing of iMIL-88A/PAN/PVP NFM. Furthermore, the intensity of the peak at 10.2° in the XRD pattern of pMIL-88A/PAN NFM was higher than that of iMIL-88A/PAN/PVP NFM, indicating that MIL-88A particles became exposed after washing and a more regular MOF crystal morphology was formed. The above results confirmed the necessity of PVP as a pore forming sacrificial agent, and demonstrated the successful preparation of the pMIL-88A/PAN NFM.

The porosity of the as-fabricated NFMs, which is critical for dye adsorption and removal, was evaluated using N_2_ adsorption–desorption experiments. As shown in Figure 3d, the Brunauer–Emmett–Teller (BET) surface area of the pMIL-88A/PAN NFM was calculated to be 143.21 m^2^ g^−1^, which is 3–3.5 times that of MIL-88A powder and pPAN NFM (46.89 and 41.57 m^2^ g^−1^). Moreover, compared to MIL-88A powder and pPAN NFM, the pMIL-88A/PAN NFM showed a high content of pore size distribution in the range of 2–30 nm (Figure S5). These results indicate that the combinations of MIL-88A and porous PAN nanofibers generated abundant mesoporous structures, which were attributed to the formation of MOF–polymer interfaces. Therefore, pMIL-88A/PAN NFM with a good porous structure is expected to achieve efficient dye adsorption and removal.

### 2.2. Adsorption Performance of the NFMs

#### 2.2.1. Dye Removal Efficiencies

**Figure 4:**
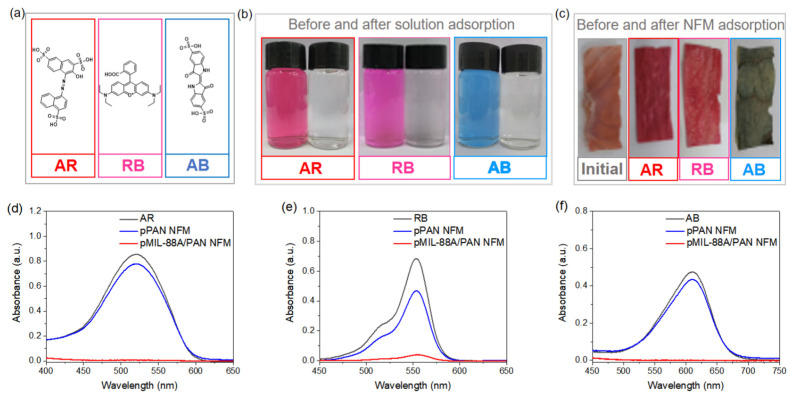
(**a**) Chemical structures of AR, RB, and AB. (**b**) Photographs of dye solutions before and after adsorption. (**c**) Photographs of pMIL-88A/PAN NFMs before and after adsorption. UV−vis absorption spectra of AR (**d**), RB (**e**), and AB (**f**) solutions before and after adsorbed using pPAN and pMIL-88A/PAN NFMs.

To evaluate the adsorption performance of the as-fabricated pMIL-88A/PAN NFM for organic dyes, amaranth red (AR), rhodamine B (RB), and acid blue (AB) were used as pollutants. These are the most commonly used dyes in industry and are frequently detected in water environments. The chemical structures of the three dyes are shown in Figure 4a. The adsorption performance of the pMIL-88A/PAN NFM was evaluated by removing the dyes from wastewater samples. Figure 4b shows photographs of the dye solutions before and after adsorption and Figure 4c shows the photographs of the NFMs before and after adsorption. The quantified adsorption capacities of the NFMs for AR, RB, and AB were recorded by UV–vis absorption measurements (Figure 4d–f). The ratio of the maximum absorption peak intensity of the solution after and before adsorption can be defined as the dye removal efficiency. The dye removal efficiencies of AR, RB, and AB using pMIL-88A/PAN NFM were calculated as 99.2, 94.4, and 99.8%, respectively. Accordingly, the colors of the three dye solutions became transparent after filtration using the pMIL-88A/PAN NFM, and the colors of the relevant NFMs changed from the initial orange to the corresponding colors of the removed dyes (Figure 4b,c). However, after filtration using the pPAN NFM, the dye removal efficiencies for AR, RB, and AB were calculated as 9.0, 31.8, and 8.6%, respectively, demonstrating that pPAN NFM exhibited limited dye adsorption and removal ability. These results verify that the pMIL-88A/PAN NFM can highly effectively remove organic dyes from aqueous solutions.

#### 2.2.2. Adsorption Kinetics of pMIL-88A/PAN NFM

**Figure 5:**
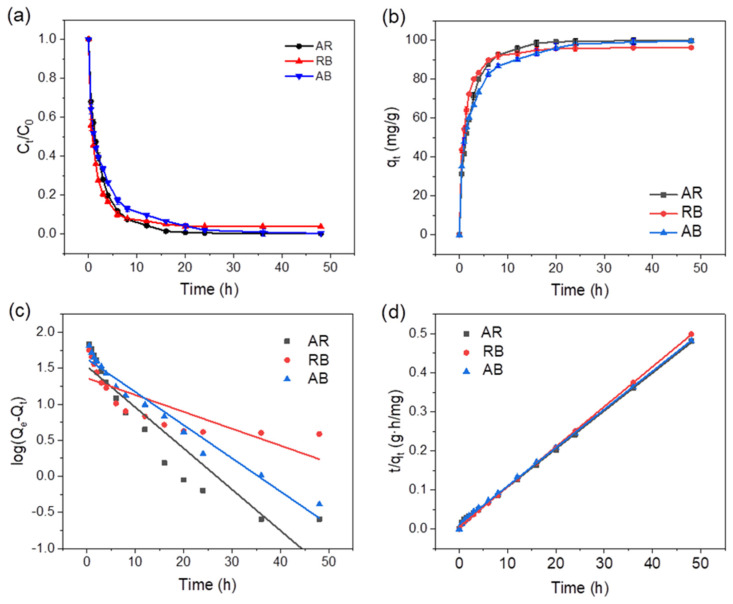
Dye removal rates (**a**) and adsorption kinetics (**b**) of pMIL-88A/PAN NFM. The average fits under pseudo−first (**c**) and pseudo−second−order (**d**) kinetic models for the adsorption of AR, RB, and AB using pMIL-88A/PAN NFM.

As shown in Figure 5a,b, due to the abundant adsorption sites of pMIL-88A/PAN NFM, the adsorption rates of the dyes were fast in the initial stages and then started to slow down until equilibriums were reached. The experimental conditions were as follows: *C*_0_ = 20 mg L^−1^ and adsorbent dosage = 0.2 mg mL^−1^. The kinetic data were subsequently fitted using the most widely used kinetic models: the pseudo-first- and pseudo-second-order kinetic models (Figure 5a and b, respectively). The linear equations of the pseudo-first- and pseudo-second-order kinetic models can be expressed as follows [47]:

(1) $$\log\left(q_{e}-q_{t}\right)=\log q_{e}-\frac{k_{1}t}{2.303}$$

(2) $$\frac{t}{q_{t}}=\frac{1}{k_{2}q_{e}2}+\frac{t}{q_{e}}$$

where *q_t_* and *q_e_* (mg g^−1^) are the adsorption capacity at time *t* and at equilibrium, respectively, and *k*_1_ (h^−1^) and *k*_2_ (g (h mg)^−1^) are the pseudo-first- and pseudo-second-order model rate constants, respectively. The fitting results are listed in Table 1. The *R*^2^ values revealed that the adsorption processes were better described by the pseudo-second-order kinetic model. Accordingly, the *q_e_s* for the adsorption of AR, RB, and AB using pMIL-88A/PAN NFM were calculated as 102.56, 97.56, and 101.94 mg g^−1^, respectively, under the pseudo-second-order kinetic model, which is 2.4–4.2 times that of the results calculated with the pseudo-first-order model (23–43 mg g^−1^).

The dye removal rates for the adsorption of AR, RB, and AB using the pPAN and pMIL-88A/PAN NFMs, and pure MIL-88A powder (Figure S6) indicate that the removal rates of the pMIL-88A/PAN NFM for the three dyes were higher than that of pure MIL-88A powder and pPAN NFM. This was attributed to the synergistic effect of the mesopores and MIL-88A particles morphology of the pMIL-88A/PAN NFM increasing the adsorption efficiency of the composite membranes for the dyes.

#### 2.2.3. Adsorption Isotherms of pMIL-88A/PAN NFM

**Figure 6:**
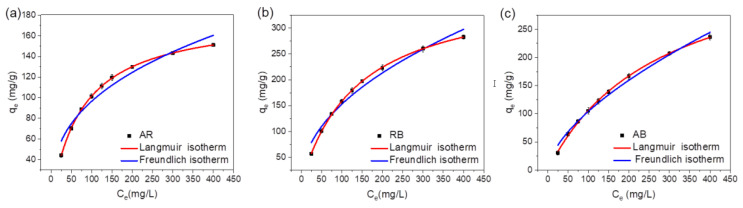
Adsorption isotherms of pMIL-88A/PAN NFM for AR (**a**), RB (**b**) and AB (**c**).

The adsorption performance of the pMIL-88A/PAN NFM was further evaluated using adsorption isotherms, and the results are shown in Figure 6 and Table 2. The classical isotherm models: Langmuir and Freundlich were used to fit the experimental data, and the corresponding linear equations are as follows [48]:

Langmuir isotherm (homogeneous and monolayer adsorption):

(3) $$\frac{C_{e}}{q_{e}}=\frac{C_{e}}{q_{m}}+\frac{1}{bq_{m}}$$

Freundlich isotherm (heterogeneous and multilayer adsorption):

(4) $$\log q_{e}=logK_{F}+\frac{1}{n}logC_{e}$$

Here, *q_e_* is the equilibrium adsorption capacity (mg g^−1^), *C*_e_ is the equilibrium concentration (mg L^−1^), *q_m_* and *b* are Langmuir constants related to the maximum adsorption capacity and binding energy, respectively, and *K_F_* and *n* are the Freundlich constant and heterogeneity factor, respectively. The fitting data are summarized in Table 2. Upon analyzing the nonlinear or linear fitting curves (Figure 6) and comparing the *R*^2^ values for the Freundlich and Langmuir models, it was concluded that the AR, RB, and AB adsorption by the pMIL-88A/PAN NFM fit the Langmuir model better. These results indicate that adsorption was a monolayer process, and specific homogeneous sites were present in the adsorbent. Therefore, the maximum adsorption capacities (*q*_max_*s*) of the pMIL-88A/PAN NFM for AR, RB, and AB were determined using the Langmuir model, and the results are summarized in Table 2. Meanwhile, adsorption isotherms of MIL-88A powder, iMIL-88A/PAN and bMIL-88A/PAN NFMs for three dyes were performed (Figure S7), and the corresponding *q*_max_*s* are listed in Table S1. The *q*_max_*s* of pure MIL-88A for AR, RB, and AB were 114.03, 119.30, and 309.45 mg g^−1^, respectively. In contrast, the *q*_max_*s* of the pMIL-88A/PAN NFM for AR, RB, and AB were 180.41, 382.75, and 399.35 mg g^−1^, respectively. The *q*_max_*s* of the pMIL-88A/PAN NFM for the three dyes, especially for RB, were higher than those of pure MIL-88A powder. In addition, the *q*_max_*s* of the iMIL-88A/PAN and bMIL-88A/PAN NFMs for the three dyes were substantially lower than those of pure MIL-88A and pMIL-88A/PAN NFM, which should be attributed to the fact that the MOF particles are wrapped in the iMIL-88A/PAN and bMIL-88A/PAN NFMs. These results indicate that the in situ electrospun and PVP removing preparation strategy of pMIL-88A/PAN NFM can expose more adsorption sites of MIL-88A for dyes, and more pore structures suitable for dyes adsorption were formed, improving its dye adsorption performance.

#### 2.2.4. Recyclability of the pMIL-88A/PAN NFM

**Figure 7:**
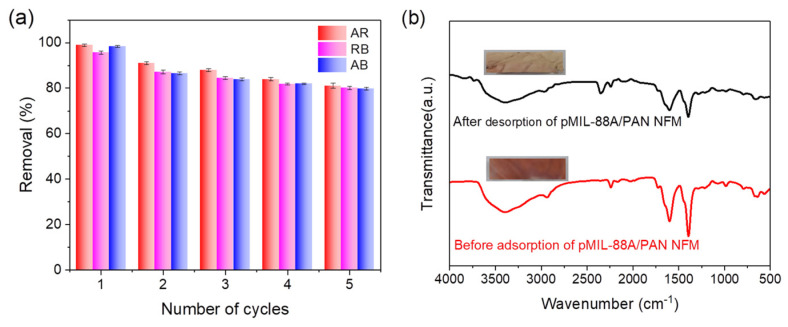
(**a**) Adsorption recyclability of the pMIL-88A/PAN NFM. (**b**) FT−IR spectra of pMIL-88A/PAN NFM before RB adsorption and after desorption of RB; the inset images are the optical photographs of the corresponding NFMs.

Adsorption–desorption experiments were conducted to evaluate the recyclability of the pMIL-88A/PAN NFM. In the first adsorption test, the pMIL-88A/PAN NFM showed 99–100% removal efficiencies for AR, RB, and AB dyes (Figure 5). Then, the dye-adsorbed NFM was treated with ethanol to destroy the interactions between MOFs and organic dyes and realize the desorption of dyes, so that the NFM can be used for the following recyclability experiments. The desorption efficiency was calculated as the ratio of the mass of NFM desorbed by ethanol to the mass of the initial NFM. The desorption efficiencies of pMIL-88A/PAN NFM adsorbed AR, RB, and AB dyes were as high as 99.9%, indicating that all three dyes could achieve a good adsorption-desorption cycling. After five adsorption–desorption cycles, the removal efficiencies of the pMIL-88A/PAN NFM for AR, RB, and AB dyes remained higher than 80% (Figure 7a). After five adsorption–desorption cycles, the NFM showed good optical shape maintenance (Figure 7b). Moreover, the NFM before RB adsorption and after desorption of RB exhibited similar FT-IR spectra, suggesting the good structural stability of the NFM under long-term application. Overall, the pMIL-88A/PAN NFM exhibited a good recyclability, which enables a long service life and will be beneficial to the recovery and reuse of dyes from wastewater.

## 3. Materials and Methods

### 3.1. Materials

PAN (Mw = 200 kDa) was obtained from Shunjie Plastic Technology Co., Ltd. (Najing, China). FeCl_3_ 6H_2_O and N,N-dimethylformamide (DMF) were purchased from Tianjin Best Chemical Co., Ltd. (Tianjin, China). PVP (Mw = 130 kDa), fumaric acid (FuA), amaranth red (AR), rhodamine B (RB), and acid blue (AB) were acquired from Energy Chemical (Guangzhou, China). All chemicals were of analytical grade and were used as received without further purification.

### 3.2. Preparation of NFMs

The NFMs were prepared by electrospinning strategy. Firstly, the PAN/PVP solu-tion (solution A) was prepared by adding 1 g of PAN powder to DMF (PAN:DMF = 1:9 *wt*/*wt*) under constant stirring for 6 h to prepare a uniform and transparent mixture. Thereafter, 1 g of PVP powder was added to the PAN solution and the mixture was stirred continuously for 5 h to prepare the solution A. Next, FeCl_3_ 6H_2_O and fumaric acid were added to DMF (FeCl_3_ 6H_2_O:FuA:DMF = 1.01:2.11:10 *wt*/*wt*), and the mixture was stirred for 3 h to prepare spinning solution B. An electrospinning solution C was prepared by mixing the solution A with the as-prepared solution B.

Subsequently, the solution C was loaded into a 10 mL syringe for electrospinning with an applied voltage of 25 kV, a receiving distance of 15 cm and a flow rate of 0.4 mL h^−1^. This fiber sample was named in situ MIL-88A/PAN/PVP (iMIL-88A/PAN/PVP) NFM. The collected iMIL-88A/PAN/PVP NFM was immersed into a mixed aqueous solution containing 50% ethanol at 50 °C for 24 h to remove PVP, followed by rinsing with ethanol and water. Then the NFM was soaked in methanol for 3 d, followed by drying in a vacuum oven at 100 °C overnight to obtain the porous MIL-88A/PAN (pMIL-88A/PAN) NFM.

The PAN/PVP NFM was obtained by electrospinning solution A, and then the pPAN NFM was obtained by removing PVP of PAN/PVP NFM. For comparison, pure MIL-88A particles, in situ MIL-88A/PAN (iMIL-88A/PAN) and blended MIL-88A/PAN (bMIL-88A/PAN) NFMs were also prepared.

### 3.3. Characterization

Scanning electron microscopy (SEM) and elemental mapping measurements were performed on a Sigma500 (ZEISS) scanning electron microscope. Fourier-transform infrared (FT-IR) spectra were recorded on a Nicolet IS 10 (Thermo Fisher, Massachusetts, America) spectrometer. X-ray diffraction (XRD) characterization was performed on a Rigaku SmartLab 9 kW using Cu Kα radiation (λ = 1.5406 Å, 40 kV, and 100 mA). Nitrogen physisorption measurements were conducted to determine the surface areas and pore volumes on a Micromeritics ASAP 2460 apparatus. UV-vis spectra were recorded on an UV-3600 (Shimadzu, Kyoto, Japan) spectrophotometer.

### 3.4. Adsorption and Desorption Experiments

#### 3.4.1. Adsorption Experiments

Kinetic experiments were conducted by adding 20 mg of adsorbent to 100 mL of AR, RB, and AB solutions with initial concentrations of 20 mg L^−1^. The adsorbent dosage was 0.2 mg mL^−1^, and 2 mL aliquots were used to determine the concentrations of the dyes. For the adsorption experiments, 10 mg of adsorbent was added to 10 mL of amaranth red, rhodamine B, and acid blue solutions with concentrations in the range of 25–400 mg L^−1^. For the adsorption–desorption experiments, 20 mg of adsorbent was added to 5 mL of 4 mg mL^−1^ amaranth red, rhodamine B, and acid blue solutions and was allow to react with the dyes for 48 h. The used adsorbents were regenerated by immersing them in ethanol for 4 h to remove the dyes, followed by rinsing with ethanol and water several times and drying under vacuum. The regenerated adsorbents were reused. The dye removal efficiency of each adsorbent sample was determined using the initial adsorption capacity. After five adsorption–desorption cycles the adsorption capacity (*q* (mg g^−1^)) of each adsorbent for the dyes was calculated as follows:

(5) $$q\;\left(\mathrm{mg}/g\right)=\frac{\left(C_{o}-C_{e}\right)V}{W}$$

where *C_o_* and *C_e_* (mg L^−1^) are the initial and equilibrium concentrations of amaranth red, rhodamine B, and acid blue in the aqueous solutions, respectively, *V* (L) is the volume of the dye solution, and *W* (g) is the mass of the dry adsorbent.

#### 3.4.2. Desorption Experiments

Specifically, the adsorbed nanofiber membranes were immersed in a mixture of ethanol: water = 1:1 for 6 h, followed by washing with ethanol and water several times, and finally the nanofiber membranes were immersed in methanol solution for 12 h, followed by vacuum drying.

## 4. Conclusions

In summary, we developed a direct electrospinning method to prepare porous MOF/polymer NFMs. MIL-88A MOF particles were formed inside the PAN/PVP fibers in situ during electrospinning, and the pMIL-88A/PAN NFM can be obtained after removing PVP by ethanol and water washing. We demonstrated that the pMIL-88A/PAN NFM achieved high dye removal efficiencies of 99.2, 94.4, and 99.8% for amaranth red, rhodamine B, and acid blue, respectively. Moreover, the pMIL-88A/PAN NFM also presented high adsorption capacities, fast adsorption kinetics, and high cycling stabilities during the processes of dye adsorption and removal. Our results demonstrated that the in situ electrospun porous MOF/polymer NFMs present promising application potential in water treatment for organic dyestuff removal.

## Figures and Tables

**Table 1:** Calculated results of pseudo-first- and pseudo-second-order constants for the adsorption of AR, RB, and AB using pMIL-88A/PAN NFM.

Dye	Pseudo-First-Order Model			Pseudo-Second-Order Model		
	*q_e_* (mg g^−1^)	*k*_1_ (h^−1^)	*R* ^2^	*q_e_* (mg g^−1^)	*k*_2_ (g (mg h)^−1^)	*R* ^2^
AR	33.81	0.1329	0.8646	102.56	0.009564	0.9992
RB	23.14	0.0546	0.6184	97.56	0.01846	0.9998
AB	42.88	0.1073	0.9626	101.94	0.008094	0.9992

**Table 2:** Calculated results of the Langmuir and Freundlich models for the adsorption of AR, RB, and AB by the pMIL-88A/PAN NFM.

Dye	Langmuir Isotherm			Freundlich Isotherm		
	*q*_max_ (mg g^−1^)	*b* (L mg^−1^)	*R* ^2^	*K_F_*	*n*	*R* ^2^
AR	180.41	0.0129	0.99989	17.8564	2.729	0.94561
RB	382.75	0.0071	0.99988	16.6871	2.079	0.97013
AB	399.35	0.0036	0.99950	6.10945	1.711	0.98950

## Data Availability

Not applicable.

## Supplementary Materials

The following supporting information can be downloaded at: https://www.mdpi.com/article/10.3390/molecules28020760/s1. The Supporting Information is available free of charge at https://pubs.acs.org/. Preparations of pure MIL-88A powder, in situ MIL-88A/PAN (iMIL-88A/PAN) and blended MIL-88A/PAN (bMIL-88A/PAN) NFMs; Figure S1: SEM images of the PAN/PVP, pPAN; Figure S2: SEM images of the pMIL-88A/PAN NFMs; Figure S3: EDS spectrum of pMIL-88A/PAN NFM; Figure S4: Pore size distributions; Figure S5: Dye removal rates of pPAN NFM, pMIL-88A/PAN NFM, and pure MIL-88A; Figure S6: Adsorption isotherms of MIL-88A powder, iMIL-88A/PAN and bMIL-88A/PAN NFMs; Table S1: Calculated results of the Langmuir and Freundlich models for the adsorption of AR, RB, and AB by MIL-88A powder, iMIL-88A/PAN and bMIL-88A/PAN NFMs. (PDF).
