# Supplementary material for: In Situ Electrospun Porous MIL-88A/PAN Nanofibrous Membranes for Efficient Removal of Organic Dyes
Source: Molecules. 2023 Jan 12;28(2):760. doi: 10.3390/molecules28020760 (PMC9860898; doi:10.3390/molecules28020760)
Supplement: Supplementary file 1 [file molecules-28-00760-s001.zip › molecules-2163696-supplementary.pdf]

Supplementary Materials

In situ electrospun porous MIL-88A/PAN nanofibrous membranes for efficient removal of organic dyes

Preparation of pure MIL-88A powder

MIL-88A powder was synthesized according to a previously reported method[S1]. Typically, $FeCl_3 \cdot 6H_2O$, Fumaric acid, and N-N dimethylformamide were added to a 100 ml PTFE lined autoclave. The autoclave was covered and kept at 100 °C for 24 h to obtain a red crystal. The synthesized MOF crystals were extracted in DMF and ethanol for 12 h, and then dried under vacuum at 90 °C for 24 h to obtain MIL-88A powder.

Preparation of in situ MIL-88A/PAN (iMIL-88A/PAN) NFM

Solution A was obtained by dissolving 1.0 g PAN in 9 ml DMF. Then 1.01 g fumaric acid was added to 10 ml DMF, and 2.11 g $FeCl_3 \cdot 6H_2O$ was added after dissolution, and B liquid was obtained after stirring evenly. The spinning solution was obtained by mixing liquid A and liquid B in A ratio of 1:1 (v/v). The electrospinning voltage is 25 kV, and the feed speed is 0.4 ml /h. keep a 15 cm distance between the rotating drum and the tip of the syringe. The resulting nanofiber membrane were soaked in methanol for 3 days and dried overnight at 100 °C in a vacuum oven.

Preparation of blended MIL-88A/PAN (bMIL-88A/PAN) NFM

1.0 g PAN was dissolved in 9 ml of DMF and 1.7 g MIL-88A particles were dispersed in the solution via sonication. The obtained solution was electrospun at a voltage of 25 kV with a feeding rate of 0.4 mL/h. A distance of 15 cm was applied between the rotating drum and the syringe tip. The obtained fiber membrane was soaked in methanol for 3 days and dried at 100 °C in a vacuum oven overnight.

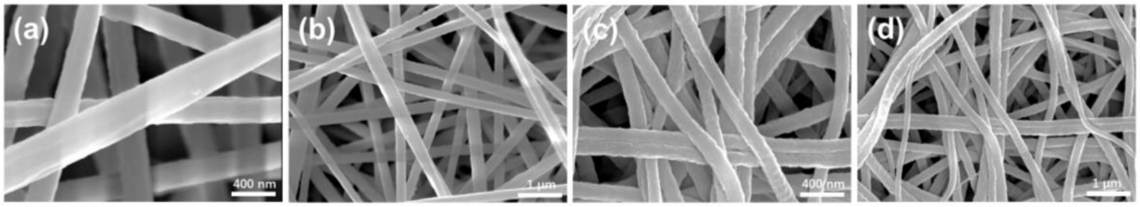

Figure S1. Scanning electron microscopy (SEM) images of the PAN/PVP NFM with different scale bars: (a) 400 nm and (b) 1 μm. SEM images of porous PAN (pPAN) NFM with different scale bars: (c) 400 nm and (d) 1 μm.

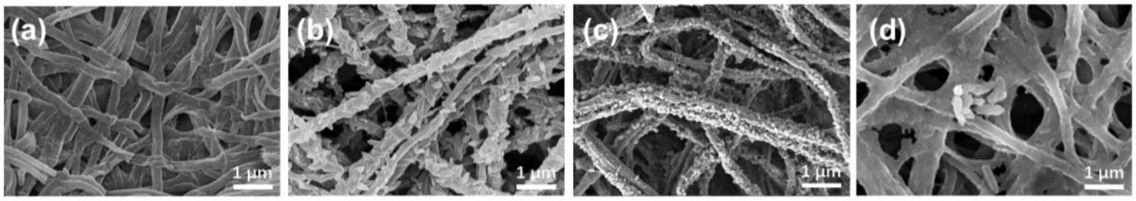

Figure S2. SEM images of the pMIL-88A/PAN NFMs under different feeding rates: (a) 0.8 mL/h, (b) 0.6 mL/h, (c) 0.4 mL/h, and (d) 0.2 mL/h.

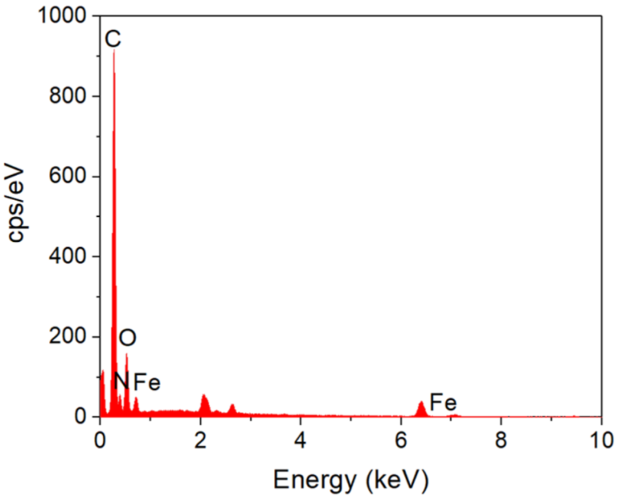

Figure S3. EDS spectrum of pMIL-88A/PAN NFM.

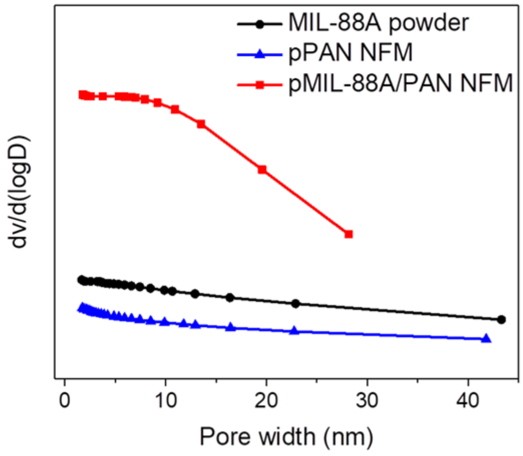

Figure S4. Pore size distributions of the MIL-88A powder, pPAN and pMIL-88A/PAN NFMs.

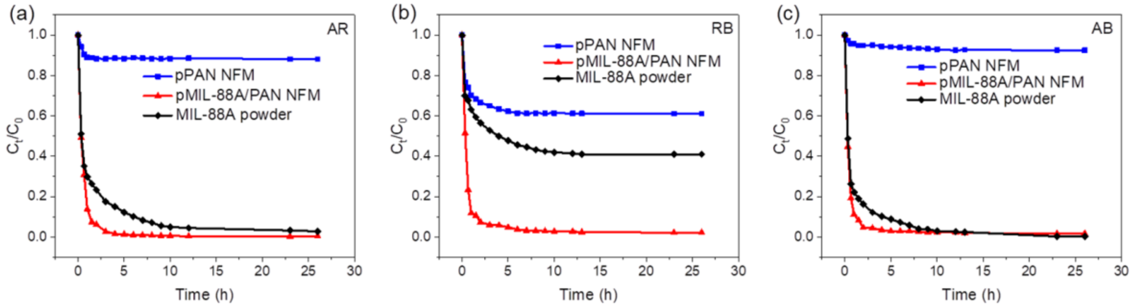

Figure S5. Dye removal rates of pPAN NFM, pMIL-88A/PAN NFM, and pure MIL-88A powder for AR (a), RB (b), and AB (c).

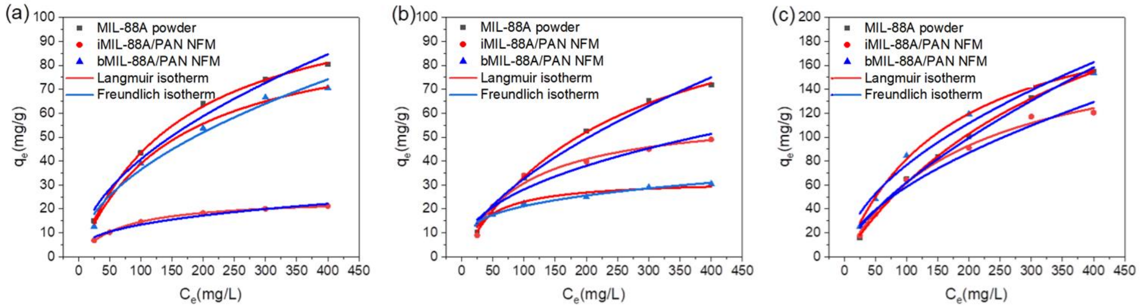

Figure S6. Adsorption isotherms of AR (A), RB (B), and AB (C) by different adsorbents (dye concentration: 25-400 mg/L, adsorbent dosage: 0.4 mg/ml)

Table S1. Calculated results of the Langmuir and Freundlich models for the adsorption of AR, RB, and AB by MIL-88A powder, iMIL-88A/PAN and bMIL-88A/PAN NFMs.

Dye	sample	Langmuir isotherm			Freundlich isotherm		
		qmax (mg/g)	b (L/mg)	R2	KF	n	R2
AR	MIL-88A powder	114.03	0.00615	0.99927	3.61509	1.90041	0.97199
	iMIL-88A/PAN NFM	24.63	0.1467	0.99906	2.57019	2.7818	0.96204
	bMIL-88A/PAN NFM	97.76	0.0061	0.99499	3.43355	1.9499	0.97303
RB	MIL-88A powder	119.30	0.00389	0.99909	2.05537	1.66589	0.97949
	iMIL-88A/PAN NFM	60.65	0.0103	0.99419	3.8093	2.3024	0.91032
	bMIL-88A/PAN NFM	32.19	0.02519	0.99735	6.0013	3.651634	0.98315
AB	MIL-88A powder	309.45	0.00249	0.99787	2.79859	1.4848	0.98956
	iMIL-88A/PAN NFM	186.75	0.00496	0.99124	4.20935	1.7495	0.96224
	bMIL-88A/PAN NFM	223.23	0.00577	0.99744	6.34243	1.84699	0.96721

Reference

[S1] Xue, B.; Du, L.; Jin, J.; Meng, H.; Mi, J. In situ growth of MIL-88A into polyacrylate and its application in highly efficient photocatalytic degradation of organic pollutants in water. APPL. SURF. SCI. 2021. 564 150404.
